# Supplementary material for: Metagenomic Insight into the Community Structure of Maize-Rhizosphere Bacteria as Predicted by Different Environmental Factors and Their Functioning within Plant Proximity
Source: Microorganisms. 2021 Jun 30;9(7):1419. doi: 10.3390/microorganisms9071419 (PMC8304108; doi:10.3390/microorganisms9071419)
Supplement: Supplementary file 1 [file microorganisms-09-01419-s001.zip › microorganisms-1275321-supplementary.pdf]

Metagenomic insight into the community structure of maize-rhizosphere bacteria as predicted by different environmental factors and their functioning within plant proximity

Saheed Adekunle Akinola[1], Ayansina Segun Ayangbenro[1], Olubukola Oluranti Babalola[1*]

1. *Food Security and Safety Niche, Faculty of Natural and Agricultural Sciences, North-West University, Private Mail Bag X2046, Mmabatho 2735, South Africa.*

Corresponding author: Olubukola Oluranti Babalola: olubukola.babalola@nwu.ac.za

Table S1. The relative abundance of Bacterial phylum in rhizosphere soil of maize and its surrounding soils

Bacterial phylum	Ls	Rs	Lc	Rc	*p*-value
Proteobacteria	42.53 ± 1.96[a]	38.30 ± 2.30[ab]	39.31 ± 1.49[ab]	35.35 ± 2.82[b]	0.05
Actinobacteria	36.27 ± 2.07[a]	41.61 ± 2.50[b]	35.18 ± 2.69[ab]	41.53 ± 2.46[ab]	0.05
Acidobacteria	5.53 ± 0.66[a]	3.40 ± 0.74[b]	6.53 ± 1.15[ab]	3.15 ± 1.65[ab]	0.03
Gemmatimonadetes	3.79 ± 0.26[ab]	1.93 ± 0.33[b]	5.36 ± 1.75[a]	2.27 ± 0.75[b]	0.01
Bacteroidetes	3.39 ± 0.54[a]	3.34 ± 0.76[a]	3.42 ± 0.57[a]	4.76 ± 0.21[a]	0.12
Firmicutes	1.88 ± 0.25[a]	2.20 ± 0.19[a]	2.13 ± 0.14[a]	2.06 ± 0.64[a]	0.68
Verrucomicrobia	1.31 ± 0.08[a]	2.05 ± 0.51[a]	1.75 ± 0.29[a]	2.10 ± 1.17[a]	0.42
Planctomycetes	1.30 ± 0.17[a]	2.26 ± 0.55[a]	1.64 ± 0.17[a]	2.16 ± 1.09[a]	0.25
Chloroflexi	1.67 ± 0.23[a]	2.06 ± 0.31[a]	1.82 ± 0.12[a]	1.88 ± 0.54[a]	0.55
Cyanobacteria	0.79 ± 0.06[a]	1.08 ± 0.22[a]	0.98 ± 0.08[a]	1.04 ± 0.51[a]	0.55
unclassified (Bacteria)	0.53 ± 0.05[a]	0.67 ± 0.13[a]	0.63 ± 0.03[a]	0.65 ±0.22[a]	0.55
Nitrospirae	0.31 ± 0.03[a]	0.32 ± 0.09[a]	0.42 ± 0.04[a]	0.27 ± 0.08[a]	0.23
Deinococcus-Thermus	0.32 ± 0.02[a]	0.34 ± 0.02[a]	0.37 ± 0.03[a]	0.36 ± 0.04[a]	0.25
Chlorobi	0.09 ± 0.01[a]	0.11 ± 0.02[a]	0.12 ± 0.02[a]	0.11 ± 0.04[a]	0.53
Spirochaetes	0.05 ± 0.00[a]	0.06 ± 0.01[a]	0.06 ± 0.01[a]	0.06 ± 0.02[a]	0.64
Aquificae	0.04 ± 0.00[a]	0.04 ± 0.01[a]	0.05 ± 0.01[a]	0.04 ± 0.02[a]	0.72
Synergistetes	0.04 ± 0.00[a]	1.05 ± 0.05[b]	0.05 ± 0.02[a]	0.05 ± 0.01[a]	<0.00
Thermotogae	0.03 ± 0.00[a]	0.06 ± 0.01[b]	0.05 ± 0.00[ab]	0.05 ± 0.01[ab]	0.04
Lentisphaerae	0.03 ± 0.00[a]	0.04 ± 0.01[a]	0.04 ± 0.01[a]	0.04 ± 0.01[a]	0.44
Candidatus Poribacteria	0.04 ± 0.00[a]	0.03 ± 0.00[ab]	0.03 ± 0.00[ab]	0.02 ± 0.01[b]	0.01
Chlamydiae	0.04 ± 0.00[a]	0.04 ± 0.00[a]	0.03 ± 0.00[a]	0.03 ± 0.01[a]	1.00
Fusobacteria	0.03 ± 0.00[a]	0.02 ± 0.00[b]	0.04 ± 0.00[ab]	0.02 ± 0.01[b]	0.05

Each value is expressed as mean ± standard deviation (n=3). [<a-z>] indicates significant difference in values of samples according to Tukey's HSD test (p≤0.05).

Table S2. The relative abundance of Bacterial class in rhizosphere soil of maize and its surrounding soils

**Bacterial class**	**Ls**	**Rs**	**Lc**	**Rc**	***p*-value**
Actinobacteria	41.98 ± 2.31^{ab}	48.08 ± 2.43^{ab}	40.27 ± 2.90^{ab}	48.07 ± 2.23^{b}	0.03
Alphaproteobacteria	20.81 ± 1.32^{a}	18.97 ± 1.63^{a}	21.04 ± 1.46^{a}	17.77 ± 0.16^{a}	0.14
Betaproteobacteria	11.51 ± 0.69^{a}	7.25 ± 1.31^{b}	8.47 ± 0.60^{b}	7.96 ± 0.74^{b}	0.01
Gemmatimonadetes	4.39 ± 0.31^{ab}	2.24 ± 0.39^{a}	6.32 ± 2.11^{b}	2.65 ± 0.97^{a}	0.02
Deltaproteobacteria	3.27 ± 0.29^{a}	4.75 ± 1.20^{a}	4.23 ± 0.67^{a}	4.72 ± 1.60^{a}	0.35
Solibacteres	3.23 ± 0.46^{ab}	2.15 ± 0.56^{a}	4.01 ± 0.82^{b}	1.98 ± 0.13^{a}	0.02
Gammaproteobacteria	3.82 ± 0.86^{a}	3.74 ± 0.54^{a}	3.07 ± 0.11^{a}	3.48 ± 0.29^{a}	0.57
Planctomycetacia	1.50 ± 0.19^{a}	2.63 ± 0.01^{b}	1.93 ± 0.21^{ab}	2.53 ± 0.36^{b}	0.00
Sphingobacteria	1.60 ± 0.30^{a}	1.22 ± 0.32^{a}	1.39 ± 0.16^{a}	1.71 ± 0.03^{a}	0.25
Acidobacteria	1.07 ± 0.15^{a}	0.56 ± 0.09^{b}	1.03 ± 0.16^{a}	0.52 ± 0.25^{b}	0.01
Bacilli	1.01 ± 0.18^{a}	1.14 ± 0.15^{a}	1.10 ± 0.06^{a}	1.16 ± 0.35^{a}	0.82
Clostridia	0.77 ± 0.07^{a}	0.93 ± 0.08^{a}	0.92 ± 0.07^{a}	0.80 ± 0.32^{a}	0.53
Verrucomicrobiae	0.62 ± 0.06^{a}	0.85 ± 0.26^{a}	0.82 ± 0.12^{a}	0.86 ± 0.55^{a}	0.72
Chloroflexi	0.79 ± 0.15^{a}	1.11 ± 0.24^{a}	0.90 ± 0.10^{a}	1.04 ± 0.31^{a}	0.35
Cytophagia	0.49 ± 0.05^{a}	0.65 ± 0.09^{a}	0.60 ± 0.02^{a}	0.87 ± 0.03^{b}	0.00
Spartobacteria	0.38 ± 0.01^{a}	0.65 ± 0.16^{a}	0.51 ± 0.08^{a}	0.67 ± 0.39^{a}	0.33
Nitrospira	0.36 ± 0.04^{a}	0.37 ± 0.11^{a}	0.50 ± 0.05^{a}	0.32 ± 0.10^{a}	0.23
Thermomicrobia	0.44 ± 0.06^{a}	0.47 ± 0.02^{a}	0.49 ± 0.00^{a}	0.43 ± 0.09^{a}	0.63
Ktedonobacteria	0.48 ± 0.03^{a}	0.36 ± 0.03^{ab}	0.49 ± 0.01^{a}	0.33 ± 0.09^{b}	0.02
Deinococci	0.38 ± 0.02^{a}	0.40 ± 0.04^{a}	0.44 ± 0.04^{a}	0.42 ± 0.06^{a}	0.44
Opitutae	0.28 ± 0.01^{a}	0.46 ± 0.15^{a}	0.38 ± 0.10^{a}	0.50 ± 0.25^{a}	0.37
Flavobacteria	0.35 ± 0.06^{a}	0.41 ± 0.09^{a}	0.38 ± 0.09^{a}	0.56 ± 0.03^{a}	0.09
Bacteroidia	0.26 ± 0.02^{a}	0.29 ± 0.02^{a}	0.32 ± 0.06^{a}	0.34 ± 0.05^{a}	0.17
Chlorobia	0.11 ± 0.01^{a}	0.12 ± 0.03^{a}	0.14 ± 0.02^{a}	0.13 ± 0.06^{a}	0.76
Gloeobacteria	0.10 ± 0.01^{a}	0.11 ± 0.02^{a}	0.13 ± 0.02^{a}	0.11 ± 0.05^{a}	0.66
Negativicutes	0.07 ± 0.01^{a}	0.07 ± 0.01^{a}	0.08 ± 0.01^{a}	0.07 ± 0.03^{a}	0.87

Each value is expressed as mean ± standard deviation (n=3). <a-z> indicates significant difference in values of samples according to Tukey's HSD test ($p \leq 0.05$).

Table S3. The relative abundance of Bacterial order in rhizosphere soil of maize and its surrounding soils

**Bacterial order**	**Ls**	**Rs**	**Lc**	**Rc**	***p*-value**
Actinomycetales	38.62 ± 1.81[a]	46.71 ± 1.25[b]	37.20 ± 3.02[a]	45.89 ± 1.83[b]	0.00
Rhizobiales	10.13 ± 0.54[a]	9.59 ± 0.61[ab]	9.98 ± 0.05[ab]	7.72 ± 1.03[b]	0.02
Burkholderiales	10.49 ± 0.73[a]	6.02 ± 1.15[b]	6.79 ± 0.73[b]	6.70 ± 0.09[b]	0.00
Solirubrobacterales	5.86 ± 0.69[a]	5.72 ± 1.19[a]	5.77 ± 0.34[a]	4.78 ± 0.46[a]	0.16
Gemmatimonadales	7.37 ± 0.36[a]	2.62 ± 0.45[b]	5.06 ± 2.43[ab]	3.11 ± 1.18[b]	0.01
Sphingomonadales	5.04 ± 1.20[a]	3.54 ± 0.50[bc]	4.88 ± 0.24[ac]	3.87 ± 0.39[c]	0.01
Solibacterales	3.73 ± 0.53[a]	2.52 ± 0.68[b]	4.68 ± 0.04[ab]	2.32 ± 1.35[ab]	0.05
Myxococcales	2.65 ± 0.28[a]	4.18 ± 0.15[b]	3.48 ± 0.08[b]	4.11 ± 0.40[b]	0.00
Planctomycetales	1.74 ± 0.22[a]	3.09 ± 0.88[a]	2.26 ± 0.23[a]	2.97 ± 1.64[a]	0.30
Sphingobacteriales	1.84 ± 0.34[a]	1.42 ± 0.38[a]	1.62 ± 0.18[a]	1.99 ± 0.01[a]	0.26
unclassified (from *Acidobacteria*)	1.18 ± 0.09[ab]	0.71 ± 0.17[a]	1.46 ± 0.21[b]	0.70 ± 0.39[a]	0.02
Acidobacteriales	1.19 ± 0.17[a]	0.66 ± 0.12[b]	1.25 ± 0.18[a]	0.61 ± 0.30[ab]	0.02
Rhodospirillales	1.02 ± 0.02[a]	0.82 ± 0.11[a]	1.05 ± 0.02[a]	0.92 ± 0.17[a]	0.10
Bacillales	1.01 ± 0.19[a]	1.08 ± 0.04[a]	1.11 ± 0.08[a]	1.22 ± 0.39[a]	0.72
Chloroflexales	0.71 ± 0.14[a]	1.00 ± 0.23[a]	0.81 ± 0.10[a]	0.90 ± 0.30[a]	0.42
Caulobacterales	0.91 ± 0.13[a]	1.02 ± 0.11[a]	0.92 ± 0.07[a]	1.05 ± 0.11[a]	0.47
Verrucomicrobiales	0.71 ± 0.07[a]	1.00 ± 0.31[a]	0.96 ± 0.14[a]	1.01 ± 0.66[a]	0.69
unclassified (from Bacteria)	0.71 ± 0.06[a]	0.91 ± 0.22[a]	0.87 ± 0.04[a]	0.89 ± 0.34[a]	0.61
Rubrobacterales	0.79 ± 0.16[a]	0.97 ± 0.33[a]	0.79 ± 0.08[a]	1.05 ± 0.20[a]	0.51
Rhodobacterales	0.72 ± 0.03[a]	0.99 ± 0.15[ab]	0.80 ± 0.02[ab]	1.22 ± 0.27[b]	0.03
Clostridiales	0.65 ± 0.06[a]	0.82 ± 0.09[a]	0.79 ± 0.06[a]	0.69 ± 0.27[a]	0.43
Xanthomonadales	0.79 ± 0.15[a]	1.15 ± 0.15[b]	0.80 ± 0.07[ab]	1.21 ± 0.22[ab]	0.05
Cytophagales	0.56 ± 0.06[a]	0.77 ± 0.24[a]	0.70 ± 0.14[a]	1.02 ± 0.05[a]	0.09
Ktedonobacterales	0.55 ± 0.03[ab]	0.42 ± 0.03[ab]	0.57 ± 0.01[a]	0.39 ± 0.11[b]	0.02
Nitrospirales	0.42 ± 0.04[a]	0.44 ± 0.13[a]	0.58 ± 0.05[a]	0.37 ± 0.12[a]	0.24
unclassified (from *Spartobacteria*)	0.43 ± 0.02[a]	0.76 ± 0.19[a]	0.59 ± 0.09[a]	0.78 ± 0.47[a]	0.32
Desulfuromonadales	0.39 ± 0.03[a]	0.46 ± 0.11[a]	0.52 ± 0.06[a]	0.46 ± 0.21[a]	0.65
Pseudomonadales	1.18 ± 0.62[a]	0.58 ± 0.07[b]	0.45 ± 0.01[b]	0.48 ± 0.02[b]	<0.00
Chroococcales	0.33 ± 0.02[a]	0.39 ± 0.08[a]	0.41 ± 0.04[a]	0.39 ± 0.16[a]	0.71
Enterobacteriales	0.72 ± 0.47[a]	0.44 ± 0.08[a]	0.40 ± 0.02[a]	0.35 ± 0.01[a]	0.47

Table S4. The relative abundance of Bacterial family in rhizosphere soil of maize and its surrounding soils

Bacterial family	Ls	Rs	Lc	Rc	*p*-value
Gemmatimonadaceae	12.08 ± 0.66[a]	4.46 ± 0.67[b]	8.43 ± 3.68[ab]	5.61 ± 1.39[b]	0.01
Conexibacteraceae	9.77 ± 1.07[a]	9.80 ± 2.02[a]	9.52 ± 0.81[a]	8.84 ± 0.35[a]	0.87
Solibacteraceae	7.70 ± 0.87[a]	4.32 ± 1.17[b]	6.23 ± 1.34[ab]	4.14 ± 1.93[ab]	0.05
Bradyrhizobiaceae	8.48 ± 0.83[a]	6.69 ± 1.38[ab]	8.37 ± 1.31[b]	5.10 ± 0.16[ab]	0.05
Streptomycetaceae	7.80 ± 0.85[a]	11.35 ± 0.52[b]	6.98 ± 0.28[a]	8.41 ± 0.51[a]	0.00
Sphingomonadaceae	5.10 ± 0.27[a]	3.47 ± 0.46[b]	4.65 ± 0.35[ab]	3.86 ± 0.62[ab]	0.01
Nocardioidaceae	4.58 ± 0.43[a]	6.37 ± 0.67[ab]	4.28 ± 0.66[a]	8.06 ± 2.27[b]	0.03
Planctomycetaceae	2.80 ± 0.34[a]	5.18 ± 0.52[b]	3.59 ± 0.27[a]	5.16 ± 0.22[b]	0.00
Burkholderiaceae	9.68 ± 0.21[a]	2.89 ± 0.57[b]	4.39 ± 1.78[b]	2.72 ± 0.23[b]	0.00
Mycobacteriaceae	3.23 ± 0.26[a]	3.58 ± 0.11[a]	3.11 ± 0.19[a]	4.26 ± 1.28[a]	0.24
unclassified (from *Acidobacteria*)	1.96 ± 0.16[ab]	1.22 ± 0.29[a]	2.41 ± 0.28[b]	1.25 ± 0.56[a]	0.02
Comamonadaceae	2.74 ± 0.95[a]	2.63 ± 0.62[a]	2.26 ± 0.29[a]	3.25 ± 0.74[a]	0.62
Acidobacteriaceae	2.05 ± 0.30[a]	1.11 ± 0.19[b]	1.98 ± 0.25[a]	1.07 ± 0.40[b]	0.01
Frankiaceae	1.88 ± 0.10[a]	1.89 ± 0.08[a]	1.90 ± 0.03[a]	2.03 ± 0.42[a]	0.82
Myxococcaceae	1.27 ± 0.08[a]	1.62 ± 0.34[a]	1.63 ± 0.17[a]	1.69 ± 0.28[a]	0.27
Micromonosporaceae	2.28 ± 0.19[a]	4.53 ± 2.89[a]	2.13 ± 0.62[a]	2.66 ± 1.07[a]	0.41
Geodermatophilaceae	2.01 ± 0.30[a]	2.03 ± 0.49[a]	1.82 ± 0.33[a]	2.57 ± 1.58[a]	0.76
Caulobacteraceae	1.52 ± 0.23[a]	1.75 ± 0.19[a]	1.52 ± 0.07[a]	1.95 ± 0.06[a]	0.11
unclassified (from *Bacteria*)	1.19 ± 0.09[a]	1.56 ± 0.40[a]	1.44 ± 0.03[a]	1.61 ± 0.41[a]	0.41
Polyangiaceae	1.02 ± 0.09[a]	2.13 ± 0.74[a]	1.27 ± 0.21[a]	2.21 ± 0.66[a]	0.08
Pseudonocardiaceae	1.75 ± 0.13[a]	1.88 ± 0.17[a]	1.61 ± 0.28[a]	1.58 ± 0.18[a]	0.33
Xanthomonadaceae	1.32 ± 0.26[a]	1.97 ± 0.30[ab]	1.31 ± 0.08[a]	2.29 ± 0.02[b]	0.01
Micrococcaceae	1.69 ± 0.30[a]	1.55 ± 0.27[a]	1.43 ± 0.22[a]	3.05 ± 0.33[b]	0.00
Methylobacteriaceae	1.27 ± 0.11[a]	1.41 ± 0.09[a]	1.28 ± 0.03[a]	1.78 ± 0.61[a]	0.25
Verrucomicrobia subdivision 3	0.87 ± 0.10[a]	1.14 ± 0.36[a]	1.15 ± 0.13[a]	1.18 ± 0.70[a]	0.73
Rubrobacteraceae	1.31 ± 0.26[a]	1.66 ± 0.57[a]	1.30 ± 0.16[a]	1.98 ± 0.65[a]	0.41
unclassified (*Sphingobacteriales*)	0.82 ± 0.13[a]	1.04 ± 0.31[ab]	0.96 ± 0.22[ab]	1.72 ± 0.36[b]	0.04
unclassified (*Spartobacteria*)	0.72 ± 0.03[a]	1.30 ± 0.32[a]	0.98 ± 0.12[a]	1.39 ± 0.68[a]	0.19
Cytophagaceae	0.78 ± 0.09[a]	1.04 ± 0.31[ab]	0.95 ± 0.15[ab]	1.51 ± 0.16[b]	0.04
Rhodobacteraceae	0.99 ± 0.04[a]	1.39 ± 0.23[a]	1.08 ± 0.06[a]	1.87 ± 0.63[a]	0.07
Nitrospiraceae	0.70 ± 0.06[a]	0.75 ± 0.23[a]	0.96 ± 0.06[a]	0.68 ± 0.14[a]	0.30
Bacillaceae	0.92 ± 0.22[a]	1.03 ± 0.08[a]	0.95 ± 0.05[a]	1.38 ± 0.27[a]	0.11
Rhizobiaceae	1.09 ± 0.08[a]	3.34 ± 3.30[b]	1.01 ± 0.11[a]	1.24 ± 0.09[a]	<0.00

Ktedonobacteraceae	0.92 ± 0.05^{a}	0.72 ± 0.06^{b}	0.95 ± 0.04^{a}	0.71 ± 0.11^{b}	0.01
Chloroflexaceae	0.88 ± 0.16^{a}	1.24 ± 0.28^{a}	0.99 ± 0.14^{a}	1.18 ± 0.27^{a}	0.30

Each value is expressed as mean ± standard deviation (n=3). <a-z> indicates significant difference in values of samples according to Tukey's HSD test (p≤0.05).

Table S5. The relative abundance of Bacterial genus in rhizosphere soil of maize and its surrounding soils

Bacterial genus	Ls	Rs	Lc	Rc	*p*-value
Conexibacter	12.61 ± 0.30^{a}	13.59 ± 0.05^{b}	12.65 ± 0.20^{a}	12.27 ± 0.55^{a}	0.01
Gemmatimonas	16.03 ± 0.92^{a}	6.26 ± 1.25^{b}	10.89 ± 4.74^{ab}	7.79 ± 1.89^{b}	0.01
Burkholderia	10.50 ± 0.38^{a}	2.60 ± 0.64^{b}	4.24 ± 2.26^{b}	2.23 ± 0.18^{b}	0.00
Streptomyces	9.35 ± 1.02^{a}	14.78 ± 1.36^{b}	8.64 ± 0.40^{a}	10.97 ± 1.73^{ab}	0.00
Candidatus Solibacter	8.04 ± 0.05^{ab}	6.10 ± 0.94^{a}	10.22 ± 0.69^{b}	5.75 ± 2.65^{a}	0.03
Mycobacterium	4.17 ± 0.37^{a}	5.00 ± 0.29^{a}	4.13 ± 0.28^{a}	5.92 ± 1.81^{a}	0.16
Bradyrhizobium	3.66 ± 0.41^{a}	3.07 ± 0.87^{a}	3.53 ± 0.68^{a}	2.27 ± 0.19^{a}	0.18
Pseudomonas	2.23 ± 0.02^{a}	1.01 ± 0.14^{b}	0.80 ± 0.15^{b}	0.89 ± 0.11^{b}	<0.00
Nocardioides	2.87 ± 0.23^{a}	5.20 ± 0.80^{b}	2.94 ± 0.17^{ab}	7.23 ± 3.25^{ab}	0.05
Frankia	2.43 ± 0.10^{a}	2.64 ± 0.20^{a}	2.52 ± 0.07^{a}	2.82 ± 0.60^{a}	0.51
Candidatus Koribacter	2.30 ± 0.24^{ab}	1.31 ± 0.34^{a}	2.85 ± 0.33^{b}	1.29 ± 0.57^{a}	0.01
Geodermatophilus	2.59 ± 0.42^{a}	2.81 ± 0.56^{a}	2.42 ± 0.47^{a}	3.58 ± 2.22^{a}	0.67
Variovorax	1.16 ± 0.77^{a}	0.87 ± 0.33^{a}	0.53 ± 0.05^{a}	0.87 ± 0.18^{a}	0.60
Kribbella	2.19 ± 0.49^{a}	2.27 ± 0.39^{a}	1.79 ± 0.72^{a}	2.01 ± 0.39^{a}	0.73
Acidobacterium	1.63 ± 0.22a	1.03 ± 0.23b	1.77 ± 0.21^{ab}	1.00 ± 0.36^{ab}	0.03
Rubrobacter	1.70 ± 0.33^{a}	2.28 ± 0.64^{a}	1.73 ± 0.23^{a}	2.75 ± 0.91^{a}	0.25
Arthrobacter	1.87 ± 0.35^{a}	1.82 ± 0.41^{a}	1.62 ± 0.27^{a}	3.61 ± 0.78^{b}	0.01
Methylobacterium	1.64 ± 0.16^{a}	1.98 ± 0.21^{a}	1.70 ± 0.06^{a}	2.48 ± 0.06^{b}	0.00
Sphingomonas	1.51 ± 0.40^{a}	1.13 ± 0.07^{a}	1.48 ± 0.10^{a}	1.20 ± 0.14^{a}	0.28
Gemmata	1.32 ± 0.19^{a}	2.44 ± 0.14^{b}	1.70 ± 0.15^{a}	2.40 ± 0.15^{b}	0.00
Micromonospora	1.41 ± 0.12^{a}	2.95 ± 1.71^{b}	1.35 ± 0.36^{ab}	1.74 ± 0.63^{ab}	0.03
Amycolatopsis	1.29 ± 0.13^{a}	1.52 ± 0.41^{a}	1.19 ± 0.30^{a}	1.01 ± 0.17^{a}	0.34
Sphingobium	0.83 ± 0.37^{a}	0.52 ± 0.07^{a}	0.58 ± 0.07^{a}	0.53 ± 0.01^{a}	0.37
Ktedonobacter	1.19 ± 0.05^{a}	1.01 ± 0.13^{a}	1.26 ± 0.07^{a}	0.98 ± 0.15^{a}	0.07
Sorangium	1.31 ± 0.13^{a}	3.00 ± 1.17^{b}	1.68 ± 0.26^{ac}	3.06 ± 0.90^{bc}	0.00
unclassified (from Bacteria)	1.17 ± 0.08^{a}	1.76 ± 0.59^{a}	1.50 ± 0.02^{a}	1.79 ± 0.50^{a}	0.32
Streptosporangium	1.10 ± 0.13^{a}	1.49 ± 0.03^{b}	0.94 ± 0.04^{ac}	0.82 ± 0.01^{bc}	0.00

Chitinophaga	1.05 ± 0.18^{a}	1.46 ± 0.51^{ab}	1.27 ± 0.28^{ab}	2.37 ± 0.50^{b}	0.05
Anaeromyxobacter	0.93 ± 0.02^{a}	1.26 ± 0.26^{a}	1.22 ± 0.14^{a}	1.31 ± 0.17^{a}	0.05
Chthoniobacter	0.93 ± 0.04^{a}	1.84 ± 0.54^{b}	1.30 ± 0.15^{ab}	1.93 ± 0.93^{ab}	0.03
Bacillus	0.92 ± 0.24^{a}	1.55 ± 0.11^{b}	0.95 ± 0.04^{ab}	1.09 ± 0.32^{ab}	0.03
Rhodococcus	0.88 ± 0.04^{a}	1.30 ± 0.10^{b}	0.89 ± 0.09^{ab}	1.43 ± 0.46^{ab}	0.05
Mesorhizobium	0.78 ± 0.05^{a}	1.03 ± 0.09^{b}	0.75 ± 0.14^{ab}	0.78 ± 0.12^{ab}	0.04
Nitrospira	0.81 ± 0.07^{a}	0.96 ± 0.35^{a}	1.16 ± 0.07^{a}	0.83 ± 0.14^{a}	0.38
Catenulispora	0.74 ± 0.01^{a}	0.64 ± 0.09^{ab}	0.67 ± 0.07^{ab}	0.53 ± 0.06^{b}	0.30

Each value is expressed as mean ± standard deviation (n=3). <a-z> indicates significant difference in values of samples according to Tukey's HSD test (p≤0.05).

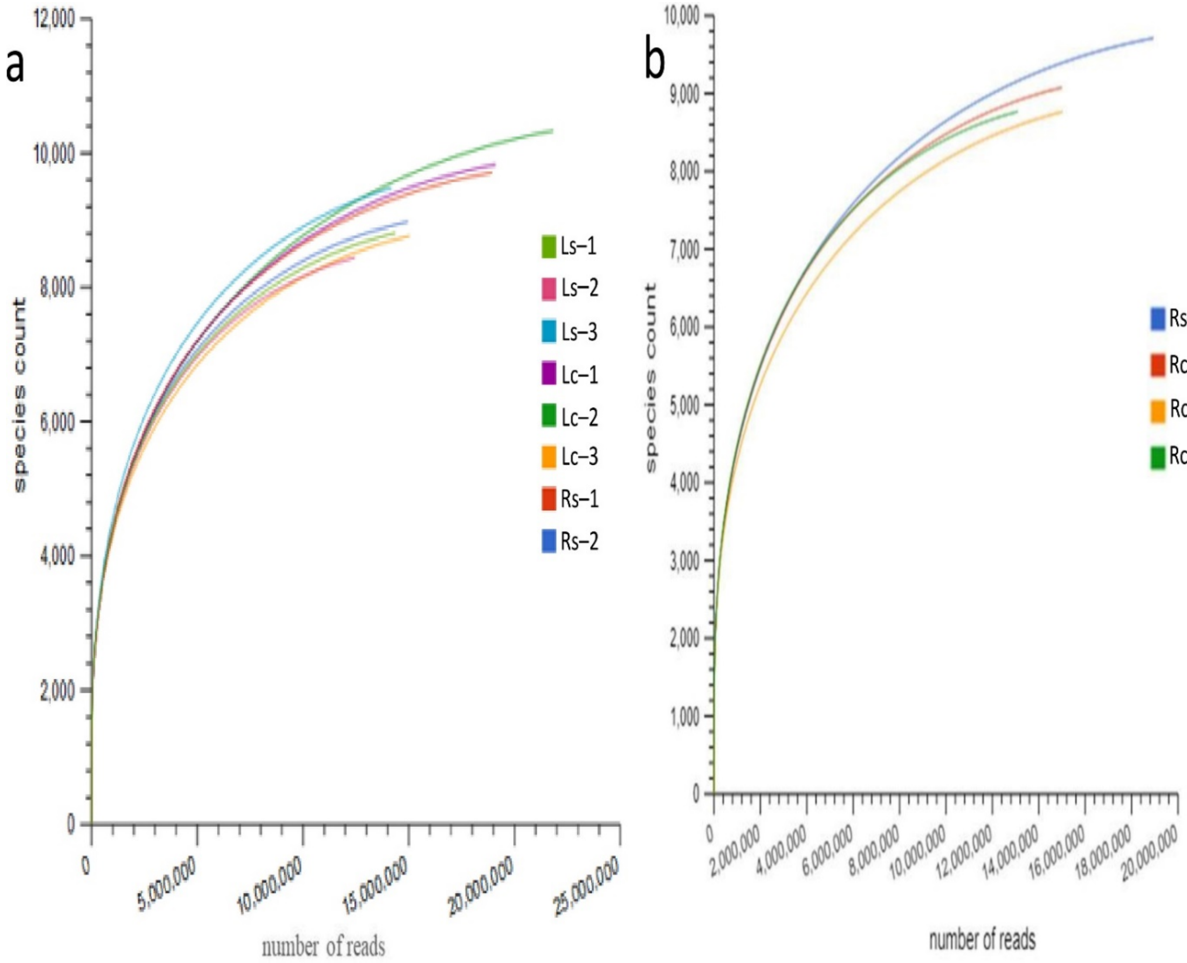

Figure S1. The richness of maize rhizosphere metagenome estimated using refraction curve. Values deduced from the mean of replicates' reads.
